# Plasmacytoid urothelial carcinoma: a rapid autopsy case report with unique clinicopathologic and genomic profile

**DOI:** 10.1186/s13000-019-0896-z

**Published:** 2019-10-21

**Authors:** Caroline T. Simon, Stephanie L. Skala, Paul D. Killen, Javed Siddiqui, Xuhong Cao, Yuanyuan Qiao, Hikmat Al-Ahmadie, Sandra I. Camelo-Piragua, Jeffrey Jentzen, Arul M. Chinnaiyan, Saravana M. Dhanasekaran, Zachery R. Reichert, Rohit Mehra

**Affiliations:** 1 Department of Pathology, University of Michigan Medical School, 2800 Plymouth Rd, Building 35, Ann Arbor, MI 48109 USA; 2 Michigan Center for Translational Pathology, Ann Arbor, MI USA; 3 Department of Pathology, Memorial Sloan Kettering Cancer Center, New York, NY USA; 4 Department of Urology, University of Michigan Medical School, Ann Arbor, MI USA; 5 Rogel Cancer Center, Michigan Medicine, Ann Arbor, MI USA; 6 Howard Hughes Medical Institute, Ann Arbor, MI USA; 7 Department of Internal Medicine, Division of Hematology and Oncology, University of Michigan Medical School, Ann Arbor, MI USA

**Keywords:** Urothelial carcinoma, Plasmacytoid, Rapid autopsy, CDH1

## Abstract

**Background:**

Rapid (“warm”) autopsies of patients with advanced metastatic cancer provide important insight into the natural history, pathobiology and histomorphology of disease in treatment-resistant tumors. Plasmacytoid urothelial carcinoma (PUC) is a rare variant of urothelial carcinoma characterized by neoplastic cells morphologically resembling plasma cells. PUC is typically aggressive, high-stage at presentation, and associated with poor outcomes. Recurrence is common in PUC, with the majority of recurrences occurring in the peritoneum.

**Case presentation:**

Here, we report rapid autopsy findings from a patient with recurrent PUC. The patient had persistent pain after cystoprostatectomy, although initial post-operative imaging showed no evidence of disease. Imaging obtained shortly before his death showed only subtle growth along vascular tissue planes; however, extensive disease was seen on autopsy. Plasmacytoid tumor cells formed sheets involving many serosal surfaces. Molecular interrogation confirmed a mutation in *CDH1* exon 12 leading to early truncation of the CDH1 protein in the tumor cells.

**Conclusions:**

The sheet-like growth pattern of PUC makes early phases of disease spread much more difficult to capture on cross-sectional imaging. Alternative forms of surveillance may be required for detection of recurrent PUC, and providers may need to treat based on symptoms and clinical suspicion.

## Introduction

Rapid (“warm”) autopsies, those performed within approximately 3 h after death, of end-stage cancer patients help us to understand the clinical and pathologic aspects of therapy-resistant disease. The goal of these autopsies is to not only establish the extent of clinical disease in the patient, but to also collect tissue from the primary tumor and metastases for molecular interrogation and comparison. Results of molecular assays performed on these tissue samples contribute to our understanding of disease progression [1]. Through genetic analysis of the patient’s primary tumor and metastases, identification of genetic mutations significant to tumor evolution including metastatic potential and treatment refractoriness is studied [2]. The majority of rapid autopsies previously reported in the literature by our group have been for primary prostatic adenocarcinoma, with one case report of metastatic renal cell carcinoma from a patient with a germline fumarate hydratase mutation [3–9]. Other institutions have reported rapid autopsies of breast and pancreatic primary tumors [10, 11]. A series of 10 rapid autopsies performed for urothelial carcinoma, including two plasmacytoid urothelial carcinomas has been presented in abstract form by Lam et al., at the American Society of Clinical Oncology meeting [12].To the best of our knowledge no dedicated case of a plasmacytoid urothelial carcinoma rapid autopsy has been so far described in the literature.

Urothelial carcinoma (UCa) is a common malignancy of the genitourinary tract [13]. Studies from The Cancer Genome Atlas (TCGA) project have demonstrated potential therapeutic targets in 69% of tumors [14]. Chromatin regulatory genes are also more frequently mutated in urothelial carcinoma than in other common cancer types, suggesting a possible therapeutic role for histone deacetylase inhibitors [14, 15]. The four mutation/focal copy number clusters were characterized by *TP53* and *RB1* mutations, *SOX4*/*E2F3* amplification, mutations in chromatin-modifying genes, and *FGFR3*, *KDM6A*, and *STAG2* mutations [15]. TCGA studies have demonstrated 5 distinct subtypes of muscle-invasive bladder cancer based on mRNA expression clustering: (1) luminal-papillary subtype (*FGFR3* mutation, fusion with *TACC3* and/or amplification, active sonic hedgehog signaling), (2) luminal-infiltrated subtype (high expression of epithelial-mesenchymal transition and myofibroblast markers, medium expression of *PD-L1* and *CTLA4*), (3) luminal subtype (high expression of luminal markers, *KRT20*, and *SNX31*), (4) basal-squamous subtype (squamous differentiation, basal keratin expression, high expression of *PD-L1* and *CTLA4*), and (5) neuronal subtype (expression of neuroendocrine and neuronal genes, and high cell-cycle signature) [15]. Most of these molecular subtypes have potential therapeutic implications.

The plasmacytoid variant of urothelial carcinoma (PUC) was first described in 1991 and is a rare histologic variant of UCa [16–18]. PUC is characterized by cells that morphologically resemble plasma cells with eccentric nuclei and eosinophilic cytoplasm arranged in sheets, cords or nests that may account the entire tumor morphology in such cases; classically, there is absent to minimal associated stromal reaction [19]. PUC is commonly locally advanced at the time of presentation, aggressive and tends to extensively involve the bladder wall, frequently extending into the perivesicular soft tissue and serosal surfaces with a high risk of local recurrence, metastatic disease and cancer-related death [20–22]. The rate of positive surgical margins in patients with PUC at the time of cystectomy has been reported to be 5 times greater than in cases of pure UCa due to the infiltration of PUC into adjacent soft tissues [23, 24]. In one single institution retrospective review, patients with PUC were more likely to receive adjuvant chemotherapy than patients with conventional UCa, given the aggressive high stage and aggressive nature of PUC [23]. However, no difference in survival has been seen in these patients who receive neoadjuvant chemotherapy compared to initial surgery [20]. The most common site of disease recurrence is the peritoneum [19]. Involvement of the perivesicular, perirectal and periureteral soft tissues is common [20–25].

A few genomic studies characterizing PUC have reported all cases to be highly aneuploid and polysomic [22]. A previous study by some of our co-authors demonstrated pathogenic *CDH1* mutations in the majority of PUC [26]. Deletions of chromosome 9p21 have been reported to play an important role and *TP53* mutations are present in a minority of PUC [22, 27]. *FGFR3* mutations have been detected in approximately 60% of cases [27]. In a recent study of 69 cases of PUC, three morphologic subtypes (classic, desmoplastic, and pleomorphic) were identified, and the desmoplastic group was found to have shortest survival (10 months) [27].

Here we report a rapid autopsy in a patient with advanced, treatment refractory plasmacytoid urothelial carcinoma, focusing on extent of metastatic disease, clinical and pathologic phenotype, molecular underpinnings and immunohistochemical profile.

## Materials and methods

Enrollment in our rapid autopsy program known as Michigan Legacy Tissue Program (MLTP) was secured, and consent for autopsy by the patient’s spouse was confirmed posthumously prior to performance of the autopsy at Michigan Medicine. The rapid autopsy protocol has been described previously [1, 6] and was followed during this autopsy. The entire gross dissection was performed simultaneously by the attending genitourinary pathologist (R.M.) and pathology residents (C.T.S. and S.L.S.). Tissues procured at the time of autopsy were placed in O.C.T. medium (Sakura Finetek USA, Torrance, CA) or formalin for frozen or permanent histologic sections, respectively. Hematoxylin and eosin (H&E), TWORT tissue gram stain, Grocott’s methenamine sliver stain and Ziehl-Neelsen were performed by the Department of Pathology at Michigan Medicine using routine laboratory methods. Immunohistochemistry by the Department of Pathology at Michigan Medicine was performed using a BenchMark ULTRA automated stainer and the ultraView Universal DAB Detection Kit (Ventana Medical Systems, Oro Valley, AZ). The following primary antibodies were used: GATA-3 (pre-dilute; Cell Marque, Rocklin, CA); CD138 (1:100, Cell Marque); CK7 (1:200; Cell Marque); CK20 (1:200; Cell Marque); CK903 (1:50, Dako, Santa Clara, CA); pancytokeratin (AE1/AE3/Cam5.2; 1:200; Chemicon/Becton Dickinson, Franklin Lakes, NJ); p53 (predilute; Ventana); PAX-8 (predilute; Cell Marque); E-cadherin (predilute; Ventana); CDX-2 (predilute; Ventana); p63 (predilute; Ventana); NKX3.1 (1:25, BioCare Medical, Pacheco, CA); PAX-2 (predilute, CellMarque); PSA (predilute, Ventana); CD10 (predilute, Ventana).

Genomic DNA was isolated from the tumor and adjacent normal tissue from the index case using the QIAamp DNA FFPE tissue kit (Cat. No./ID: 56404) according to the manufacturer’s recommended protocol. Using 50 nanograms of genomic DNA from normal and tumor samples as templated, PCR reactions (HotStarTaq DNA Polymerase - Cat No./ID: 203203) were performed (38 cycles, annealing temp. 60 °C) to amplify the 14 coding exonic regions of the *CDH1* gene (Primer sequences; Additional file 1: Table S1). 5′ end of the forward primers also contain M13 forward sequence to enable Sanger sequencing. The PCR products were first analyzed in an agarose gel to confirm the amplicon size. Subsequently, the PCR products were treated with 2 μl of ExoSAP-IT (Affymetrix P/N: 78201) for every 5 μl of PCR product and incubated first at 37 °C for 15 min, followed by 80 °C for 15 min for inactivation. Finally, the samples were diluted and submitted for Sanger sequencing (University of Michigan, DNA sequencing Core). The sequencing chromatograms assembled and analyzed by Sequencer 5.2 tool from Genecodes. CDH1 Ref seq Accession number NM_004360 was used as a reference in the analysis.

## Results

### Clinical history and sequence of events

The decedent was a 65-year-old Caucasian male with a past medical history of hypertension, environmental allergies and arthritis. His family history was significant for cancer of unknown type in his maternal grandfather, breast cancer in his mother and lung cancer in his father. Nine months prior to death the patient presented to an outside hospital emergency room with abdominal pain. Subsequent work-up included computed tomography (CT) of the abdomen and pelvis, which showed a thickened left lateral wall and dome of the urinary bladder. A biopsy at the site of CT abnormality showed muscle invasive poorly differentiated carcinoma with signet ring features, favoring urothelial primary, while a transurethral resection of bladder tumor (TURBT) showed muscle invasive high-grade UCa. The TURBT slides were not reviewed at Michigan Medicine. At that time of the biopsy a positron emission tomography (PET) scan showed no evidence of nodal disease.

The patient underwent neoadjuvant chemotherapy (3 cycles of dose-dense Methotrexate, Vinblastine, Doxorubicin and Cisplatin). PET scan after neoadjuvant chemotherapy showed persistent thickened bladder wall and mild metabolic activity. A subsequent cystoprostatectomy and bilateral inguinal lymph node dissection with ileal conduit formation was performed 5 months prior to death. The cystoprostatectomy material, which was reviewed for pathologic assessment at Michigan Medicine, showed high grade carcinoma, 7 cm per gross report, with extensive signet ring/plasmacytoid differentiation. The resection specimen demonstrated gross invasion into the perivesicular soft tissue, with a positive right perivesicular soft tissue margin. Five of six lymph nodes were positive for metastatic disease. At the time of cystoprostatectomy prostatic adenocarcinoma, Gleason score 3 + 4 = 7, Grade Group 2, with established extraprostatic extension on the right side was also identified. Given the discovery of the prostatic adenocarcinoma and lack of a surface urothelial neoplasm, immunohistochemical stains were performed on the tumor arising in the bladder, which was p63 positive (patchy) and prostate specific antigen (PSA) negative. Given these findings along with the previous history, the primary bladder disease was considered to be urothelial in origin.

Approximately 1 month after cystoprostatectomy the patient presented at Michigan Medicine to establish care. There was a clinical trial evaluating whether adjuvant atezolizumab was beneficial for high-risk localized disease (NCT02450331), but our patient was not eligible since his tumor was not predominantly conventional transitional cell carcinoma. When it was determined that he had relapsed, his Eastern Cooperative Oncology Group (ECOG) performance status had declined too much to allow therapy to be attempted and close clinical follow-up and pain management was recommended. The patient’s serum PSA was undetectable. CT of the abdomen and pelvis at 1.5 months post-operative was negative for relapse. Repeat imaging (now including chest also) at 3 months post-operative showed an area concerning for disease in the left hemi-pelvis, but no evidence of distant metastatic disease. He developed leg swelling during a long trip and was diagnosed at an outside institution with a deep vein thrombosis (DVT) and given a dose of enoxaparin and started on rivaroxaban. Nine days after treatment for DVT, the patient developed shortness of breath and presented at Michigan Medicine where he was diagnosed with pulmonary emboli of the left lower lobe and right middle lobe and a subacute DVT in the right lower extremity. The patient remained hospitalized at Michigan Medicine for 8 days, during which a PET scan showed disease progression in the pelvis with peritoneal carcinomatosis, right diaphragmatic and anterior chest wall nodules. Seventeen days prior to death the patient was admitted with worsening abdominal and scrotal swelling. CT abdomen and pelvis showed interval progression of disease and more extensive carcinomatosis. During his 2-day hospitalization he developed shortness of breath requiring a respiratory rapid response. He was transitioned to comfort care and was discharged with home hospice and comfort measures 15 days prior to death. After death the patient was transported to Michigan Medicine for participation in the rapid autopsy program.

### Pathologic findings

**Fig. 1:**
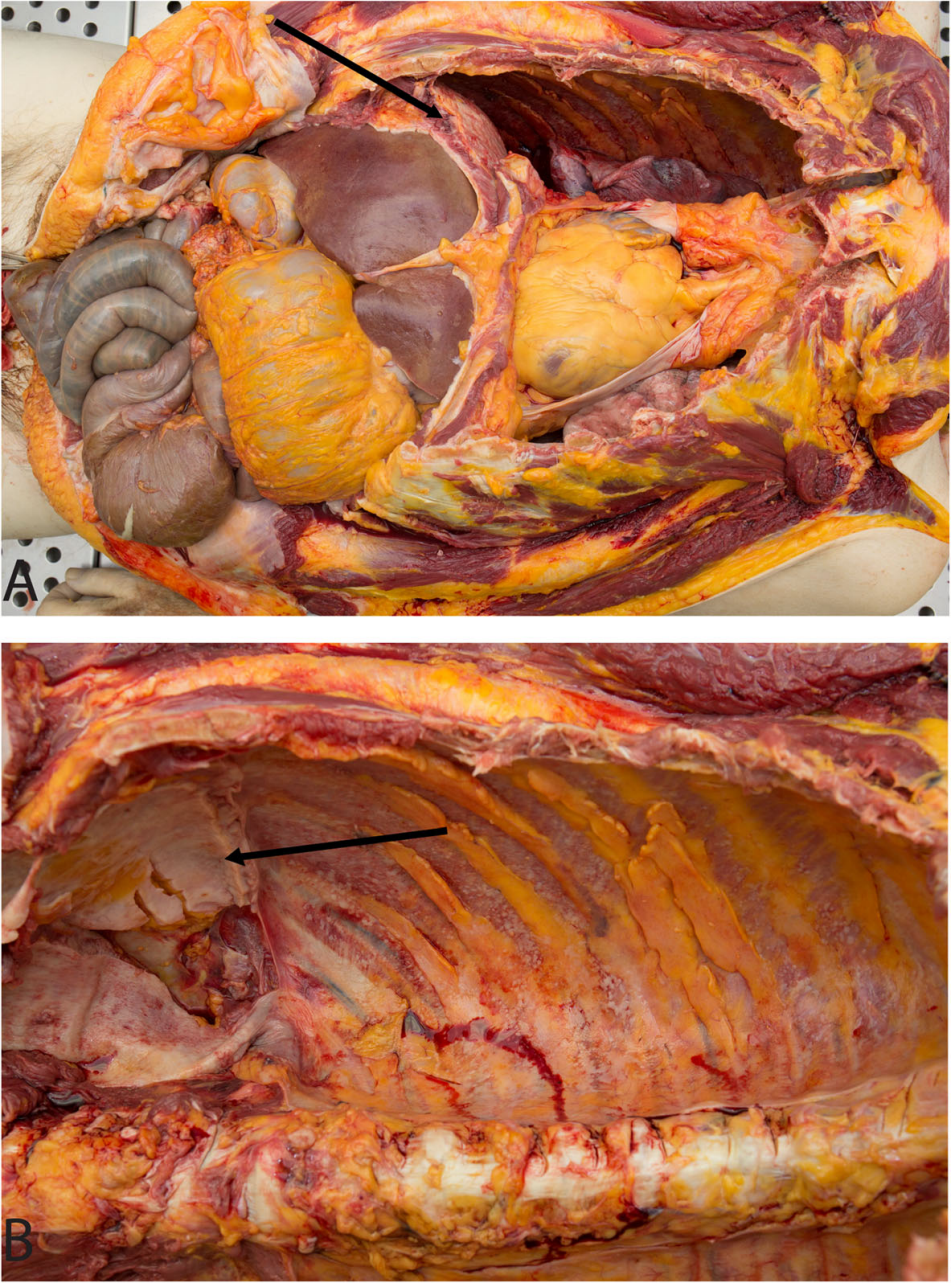
Plasmacytoid Urothelial Carcinoma in the Abdomen (in Situ). The diaphragm was diffusely thickened in a white plaque of tumor (**a**, shown before evisceration by arrow) and the peritoneal/serosal surface was diffusely thickened in a white plaque of tumor (**b**, shown after evisceration by arrow)

**Fig. 2:**
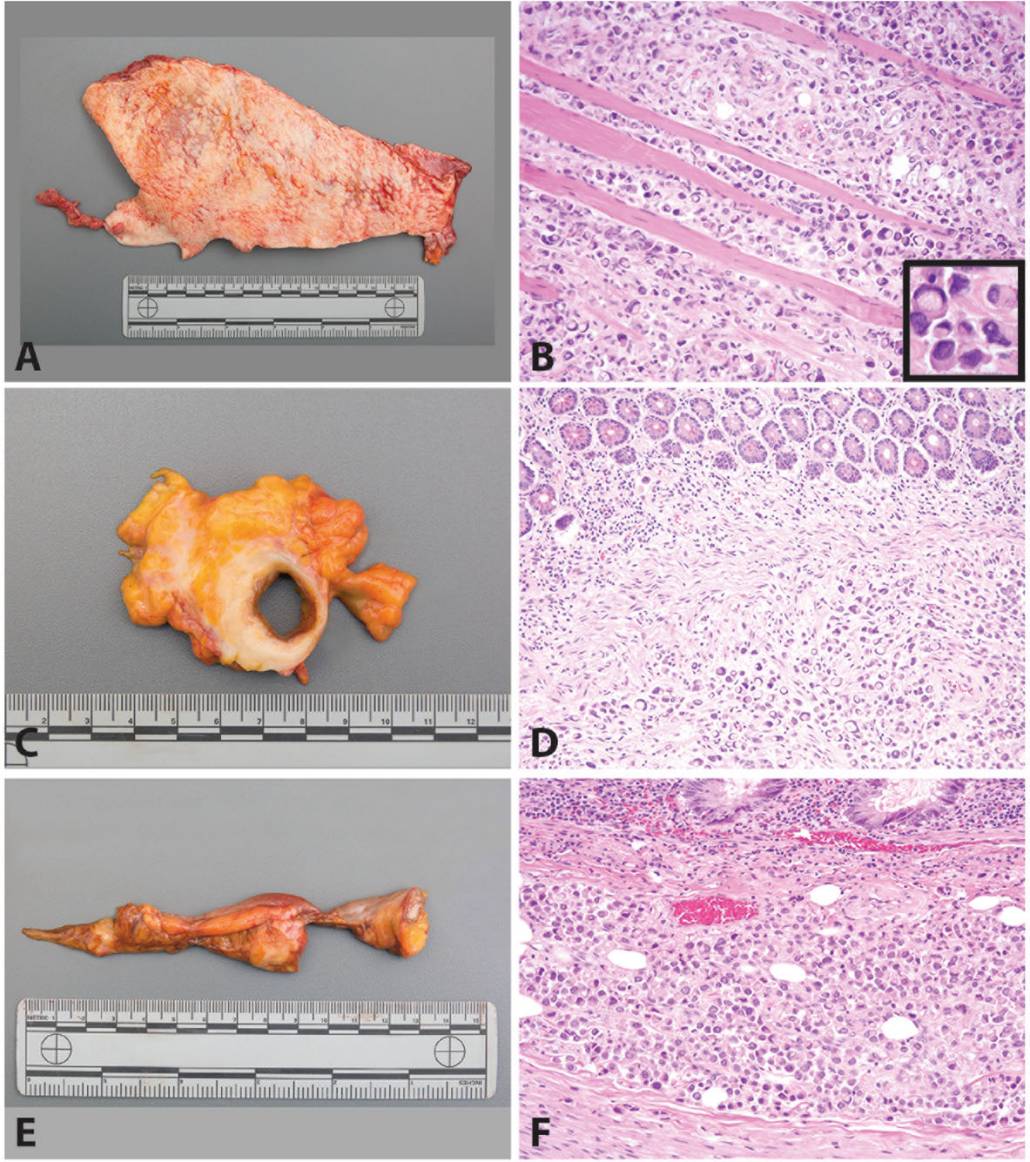
Plasmacytoid Urothelial Carcinoma in the Abdomen. The abdominal wall and peritoneum were grossly thickened with a white plaque (**a**). The skeletal muscle of the right abdominal wall was diffusely infiltrated by plasmacytoid tumor cells (**b**) [H&E 200x; inset 400x]. The lower gastrointestinal tract was diffusely involved with tumor encasing the ileum (**c**) with involvement of the submucosa (**d**) [H&E 200x] and the appendix (**e**) with tumor extending into the muscularis (**f**) [H&E 200x]. Tumor was present throughout the abdomen in the omentum, gallbladder, colon and rectum. Extra-abdominal metastases were present in the lungs, pleura, diaphragm and lymph nodes (not pictured)

The left lower lobe of the lung was adherent to the diaphragm in a thickened, white plaque. There were multiple punctate white lesions (up to 0.2 cm) on the pleural surface of the posterior aspect of the lower lobe of the right lung, right pleural cavity and diaphragm. The peritoneal/serosal surfaces were diffusely thickened by tan to white tumor (Fig. 1a, b and Fig. 2a). Microscopically, all sites of tumor showed sheets of single high-grade carcinoma cells with plasmacytoid morphology. The cells were large and round with eccentric nuclei and eosinophilic cytoplasm. A subset of cells had a signet ring-like morphology with large cytoplasmic vacuoles. Individual tumor cells were noted to infiltrate the skeletal muscle of the right abdominal wall (Fig. 2b). White-tan firm nodules and plaques diffusely involved the mesentery and omentum. The ileum (including 5 × 4 × 3 cm area of ileal conduit leading up to the stoma) (Fig. 2c), transverse colon and rectum were focally encased by white-tan tumor with gross invasion of the tumor into the muscular walls of the ileum and transverse colon. Microscopic sections confirmed that carcinoma involved the submucosa of the ileum (Fig. 2d), transverse colon and rectum. Multiple pale tan plaques involved the gallbladder serosa. The soft tissue around the appendix showed diffuse thickening, and a 2 cm tan nodule was identified adjacent to the appendix (Fig. 2e). Microscopically, carcinoma involved the muscularis of the appendix (Fig. 2f). Diffuse lymphadenopathy (up to 3 cm) in the paraesophageal, paraaortic and paravertebral regions was noted. The bilateral lungs showed extensive vascular space involvement with metastatic carcinoma and a parenchymal metastasis in the left lower lobe with extension into the left diaphragm. A 3 cm vegetation was present on the pulmonic valve; microscopically, this was composed of fibrin and karyorrhectic debris without tumor. In the area of the pancreas there was extensive tumor; no definitive normal pancreatic tissue could be identified, suggesting the pancreas was completely replaced by the neoplastic process. The spleen, liver, heart, bilateral kidneys, brain, thyroid and bone marrow were grossly not involved by tumor. The urinary bladder and prostate were surgically absent.

### Ancillary studies

Prior to coming to Michigan Medicine, microsatellite instability (MSI) testing and PD-L1 staining were performed on formalin-fixed, paraffin-embedded (FFPE) bladder tissue. The tumor was microsatellite stable, and the PD-L1 22C3 tumor proportion score was 0%. Although MSI testing is not routinely done, PD-L1 staining can be useful for selection of appropriate first line therapy in the metastatic setting. With our patient’s rapid clinical decline precluding any systemic therapy, expanded next generation sequencing was not considered to be appropriate for his clinical management.

**Fig. 3:**
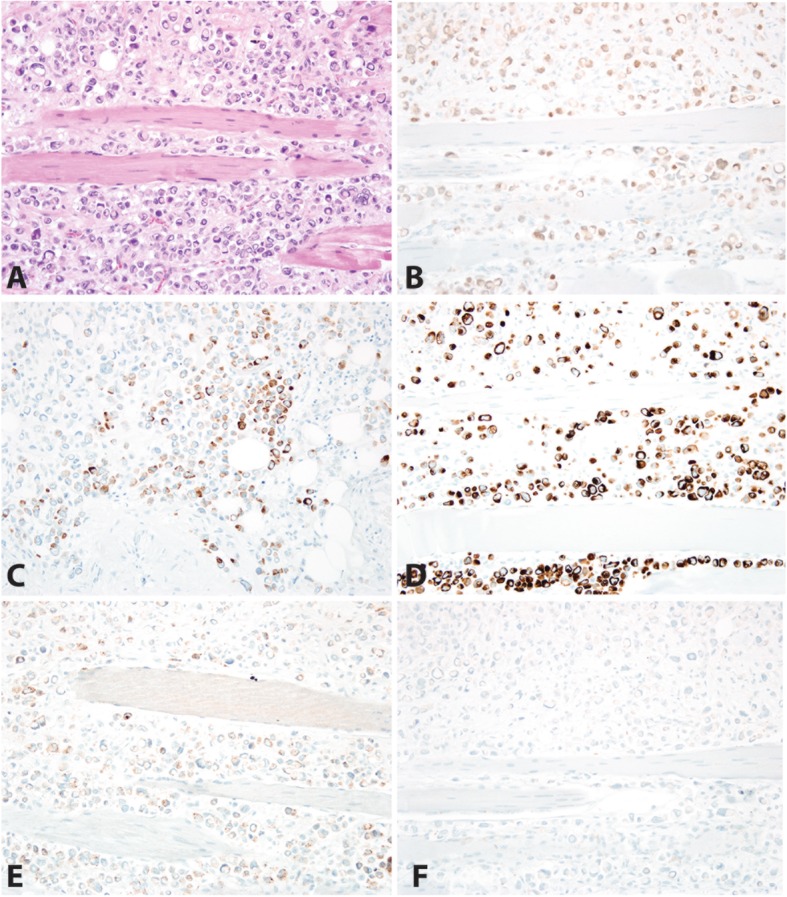
Immunohistochemical Profile of Plasmacytoid Urothelial Carcinoma in the Right Abdominal Wall. Hematoxylin and Eosin demonstrates carcinoma in sheets splaying the skeletal muscle (**a**). The neoplastic cells are diffusely positive for GATA-3 (**b**), CK20 (**c**), and CK7 (**d**). A subset of neoplastic cells is positive for CD 138 (**e**). E-cadherin is negative (**f**). Additional immunohistochemical stains show the tumor cells are diffusely positive for pancytokeratin, patchy expression of CK903, and p53, and focal expression of PAX-8 and negative for CDX-2, p63, NKX3.1, PAX-2 and PSA

Tumor cells with the plasmacytoid/signet ring-like morphology demonstrated strong expression of GATA-3 (Fig. 3a), pancytokeratin, CK7 (Fig. 3b) and CK20 (Fig. 3c), patchy expression of CD138, CK903, and p53, and focal expression of PAX-8. The tumor cells demonstrated loss of E-cadherin (Fig. 3d), with negative CDX-2, p63, NKX3.1, PAX-2, and PSA expression.

**Fig. 4:**
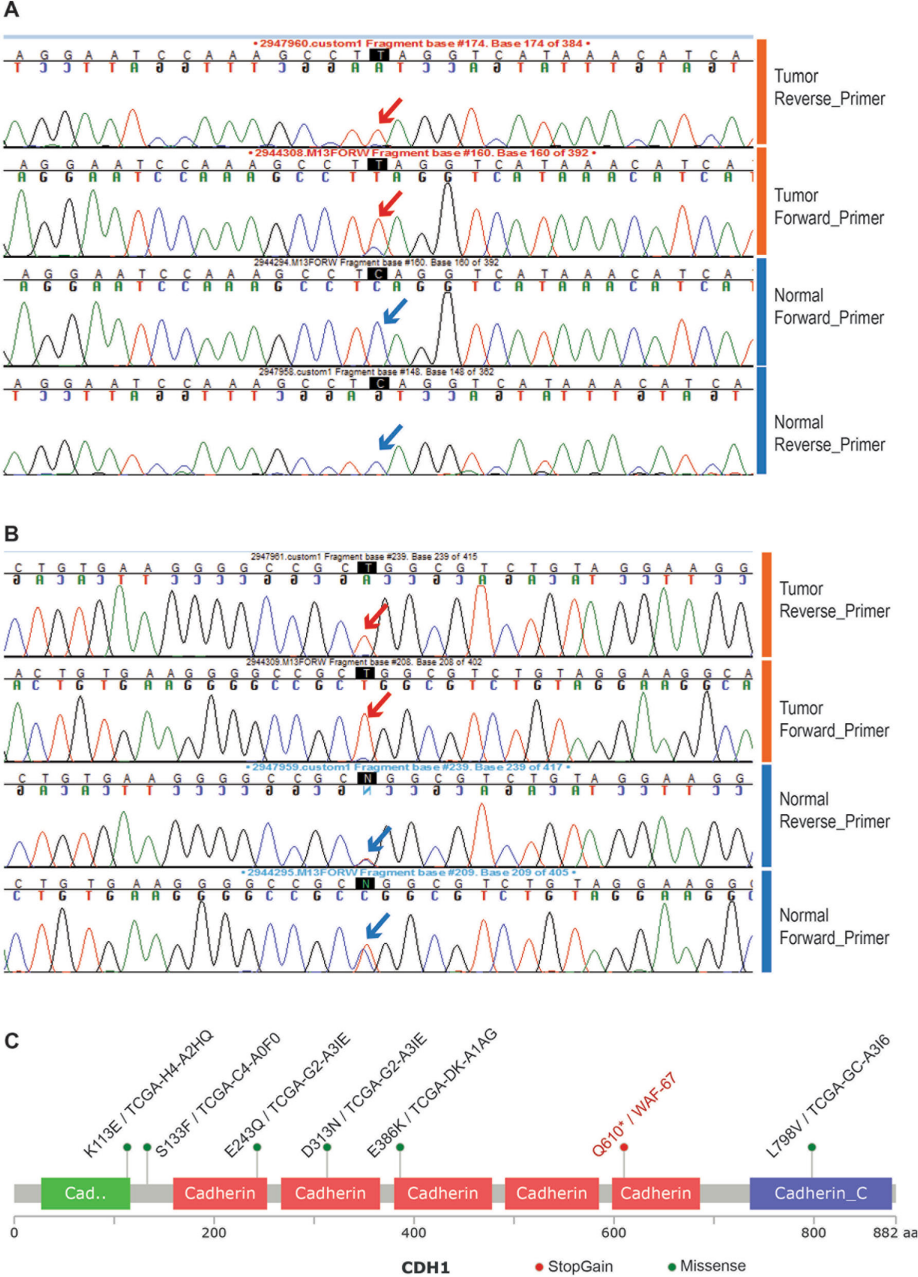
Deleterious mutation in CDH1 exon-12 in the index tumor sample. Chromatograms indicate the mutant allele present in the tumor sample (red arrows), both in the forward and reverse strand sequencing. The tumor specific mutation in exon-12; C1828T_Q610Stop gain, leads to early truncation of the CDH1 protein. The wild type allele (blue arrows) in the corresponding nucleotide position, detected in the adjacent normal sample from this index case, both in the forward and reverse strands are shown alongside for comparison (**a**). A nearby single nucleotide polymorphism (SNP) position in Exon 13 T/C2076T A692A_SNP_position (rs1801552) shows loss of heterozygosity of CDH1 loci in the tumor (**b**). Location of the deleterious stop gain mutation (red lollipop) observed in the index tumor (Sample name in red) and various missense mutations (green lollipops; of unknown significance) observed among the TCGA Bladder Urothelial Carcinomas (6/121 samples)- is represented in the CDH1 pfam protein domain structure schematic diagram using the Mutation Mapper tool (www.cbioportal.org) (**c**)

Molecular analysis performed on the index tumor demonstrated a deleterious mutation in *CDH1* exon 12 leading to early truncation of the CDH1 protein in the index tumor sample which was absent from the adjacent benign tissue (Fig. 4).

**Fig. 5:**
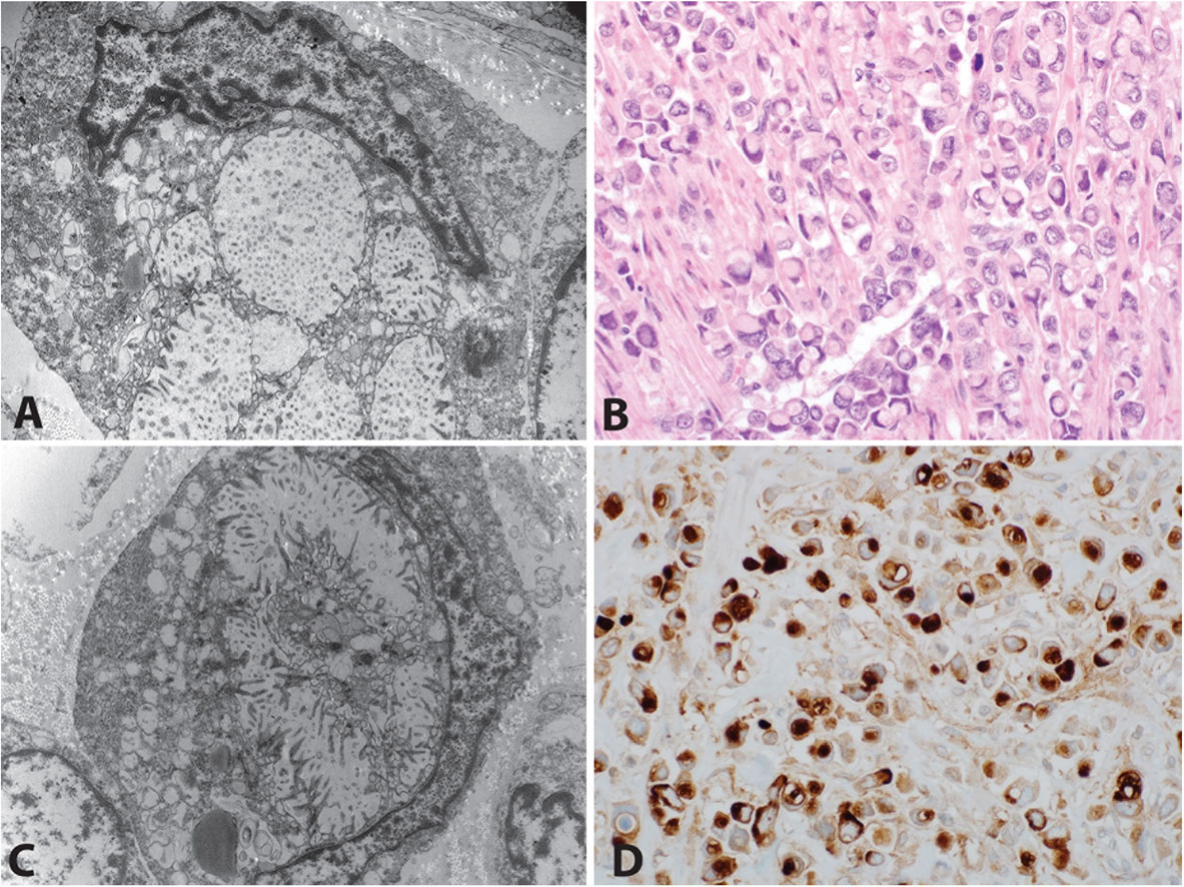
Electron microscopy analysis and CD10 immunostain of plasmacytoid urothelial carcinoma. Electron microscopy was performed on a representative section of tumor demonstrated nuclei pushed aside by intracellular vacuoles with microvilli lining the inner surface (**a**,**c**), Hematoxylin and eosin of the tumor (**b**) and a CD10 immunostain showing an apical cytoplasmic staining pattern, similar to those reported in microvillous inclusion disease (**d**)

Electron microscopic evaluation of a representative section of tumor showed nuclei pushed aside by intracellular vacuoles with microvilli lining the inner surface (Fig. 5 a, c). Given this finding reminiscent of microvillous inclusion disease, a CD10 immunohistochemical stain was performed and showed apical/luminal staining (H&E Fig. 5b, CD10 Fig. 5d) .

## Discussion

Here we describe a rapid autopsy case of advanced metastatic plasmacytoid urothelial carcinoma. To our knowledge, this is the first case report of a rapid autopsy performed for a patient with plasmacytoid urothelial carcinoma. Our patient presented with abdominal pain and was subsequently diagnosed with invasive high-grade UCa with plasmacytoid features. His treatment-resistant high-grade UCa demonstrated extensive peritoneal carcinomatosis and distant metastases involving multiple areas of the gastrointestinal tract, the biliary system, lungs, pleural surface, diaphragm, paraesophageal, paraaortic and paravertebral lymph nodes. The tumor cells displayed the classic plasmacytoid appearance with eccentric nuclei and eosinophilic cytoplasm, with a remarkable absence of any associated stromal reaction. Ancillary studies demonstrated expression of expected urothelial markers (GATA-3, pancytokeratin, CK7, CK20), patchy expression of plasma cell markers (CD138) and loss of expression for E-cadherin protein.

PUC is an aggressive variant of UCa. Similar to other reports of PUC, our patient had advanced (stage pT3b) and lymph node metastases at the time of diagnosis [28]. The plasmacytoid component of PUC varies in previous reports from 30 to 100% with the majority of cases demonstrating pure plasmacytoid morphology [19, 28]. Our case is an example of the more common pure plasmacytoid morphology with no identifiable conventional high-grade urothelial carcinoma component. Given the possibility of other malignancies which may manifest a similar morphology, it is important to rule out other tumors with plasmacytoid morphologies including plasmacytoma, signet ring cell adenocarcinoma and metastatic carcinomas from other primary sites, especially the breast and stomach [28–31]. The previously described immunophenotype of PUC reported in literature includes positive expression of CK7, 20, GATA3 and AE1/AE3, frequent expression of the plasma cell marker CD138, and absence of expression of E-cadherin in the plasmacytoid morphology [19, 27, 28, 32, 33]. Coupled with the clinical presentation of disease and pattern of spread, immunohistochemistry may play an important role in determining the primary site of disease. The immunophenotype of our case was similar to the cases previously described, with diffuse expression of urothelial markers, patchy CD138 expression, and loss of E-cadherin expression.

The presence of intracytoplasmic lumina with projecting microvilli on electron microscopy has been previously described, as has the presence of intracytoplasmic bundles of tonofilaments [34]. Additionally, the electron microscopic features of our case are similar to those described in microvillus inclusion disease, an autosomal condition causing congenital diarrhea and failure to thrive [35] that results from mutations in the *MY05B*, which encodes a cytoskeleton motor protein [36]. Microvillus inclusion disease is almost always fatal within the first 2–3 years of life [37], and there is no known increased risk of malignancy in these patients. Microvillus inclusion disease has a characteristic apical cytoplasmic staining pattern in the enterocytes with CD10 immunostain [38]. Given the similarity of ultrastructural findings between our case and reported cases of microvillus inclusion disease, a CD10 stain was performed and showed a similar pattern to microvillus inclusion disease. Given these similarities, further studies may be warranted to examine the possibility of a genetic relationship between microvillus inclusion disease and PUC.

Similar to other cases that have been described, our patient had recurrence with extensive peritoneal carcinomatosis and involvement of the perirectal soft tissue. In its earliest form, this PUC carcinomatosis manifests in a “sheet” like growth pattern rather than “nodular”- similar to its molecular counterpart, hereditary diffuse gastric cancer [39]. This makes early phases of disease spread much more difficult to capture on cross-sectional imaging, making clinical surveillance for relapse harder and potentially requiring a different staging approach (i.e. peritoneal washings and/or PET). Unfortunately, this patient suffered secondary to this challenge. His pain after surgery never improved (likely from residual disease affecting pelvic nerves), while 6-week post-operative imaging was negative for recurrence. His 3-month postoperative imaging only revealed subtle growth along vascular tissue planes on coronal slices adjacent to the left common iliac and left internal iliac vessels, yet shortly after his autopsy revealed extensive involvement of the gastrointestinal tract, submucosa of the rectum, and muscular invasion of the appendix, transverse colon and ileum, as well as extensive disease involving the pleura, lung parenchyma, diaphragm and gallbladder. This type of encasing growth has also been reported in the duodenum, with involvement of the submucosa [33].

For this variant histology, the treating provider must maintain vigilance and understand the inherent limitations in diagnosing relapse of this disease. Alternative forms of surveillance (e.g. PET) may potentially be required, and providers may need to treat based on symptoms and clinical suspicion. Given the advanced stage and aggressive behavior of PUC, prognosis is poor. Our patient died of disease 9 months after his initial diagnosis, which is similar to other reports [19, 20, 28]. Finally, our patient’s tumor demonstrated a deleterious mutation in *CDH1* exon 12 leading to early truncation of the CDH1 protein, similar to the findings of our previous study [26]. Patients with hereditary diffuse gastric cancer (germline *CDH1* mutation) undergo prophylactic gastrectomy due to the challenge in early diagnosis of this type of carcinoma [39]. Standard imaging and intraoperative frozen sections often underestimate disease burden for both diffuse gastric cancer and plasmacytoid urothelial carcinoma. PUC was excluded from the original studies showing benefit of neoadjuvant chemotherapy for these reasons, as well as its rarity. Although multiple retrospective studies have attempted to evaluate the benefit of chemotherapy for PUC, there is information about a limited number of patients and retrospective studies have inherent biases. Metastatic conventional urothelial carcinoma can be treated with immunotherapy either post-platinum chemotherapy or as a first-line in cisplatin-ineligible patients with tumors showing high PD-L1 staining. Again, PUC was excluded from the clinical trials, and to our knowledge, there are no specific reports about the responsiveness of PUC to PD1 or PD-L1 blockade. Because cisplatin-based chemotherapy has significant risks and the benefit is unclear, early cystectomy is our recommended approach for PUC management.

## Conclusions

In summary, we present rapid autopsy findings from a unique patient with extensively metastatic treatment resistant PUC. As we continue to expand our rapid autopsy program (MLTP) we will aim to include more patients with this rare variant of UCa and other genitourinary malignancies allowing for opportunities to further understand the tumorigenesis and malignant spread, locally aggressive versus distant metastases and valuable clinical correlations with molecular annotations.

## Supplementary information

**Additional file 1: Table S1.** Primer sequences for amplification of coding exonic regions of the CDH1 gene.

## Data Availability

Not applicable.

## Abbreviations

CT: Computed tomography
DVT: Deep vein thrombosis
H&E: Hematoxylin and Eosin
MLTP: Michigan Legacy Tissue Program
PET: Positron Emission Tomography
PSA: Prostate specific antigen
PUC: Plasmacytoid Urothelial Carcinoma
TCGA: The Cancer Genome Atlas
TURBT: Transurethral resection of bladder tumor
UCa: Urothelial Carcinoma
