# Supplementary material for: Plasmacytoid urothelial carcinoma: a rapid autopsy case report with unique clinicopathologic and genomic profile
Source: Diagn Pathol. 2019 Oct 21;14:113. doi: 10.1186/s13000-019-0896-z (PMC6802321; doi:10.1186/s13000-019-0896-z)
Supplement: Supplementary file 1 — Additional file 1: Table S1. Primer sequences for amplification of coding exonic regions of the CDH1 gene. [file 13000_2019_896_MOESM1_ESM.docx]

**Additional file 1: Table S1**

| S.No | Primer Name | Sequence | Tm (^0^C) |
| --- | --- | --- | --- |
| 1 | CDH1_Ex3-F | GTAAAACGACGGCCAGTTCGCTCTTTGGAGAAGGAATG | 66 |
| 2 | CDH1_Ex3-R | AAATGTCAACGGTACCAAGGC | 55.8 |
| 3 | CDH1_Ex4-F | GTAAAACGACGGCCAGTCTGTACACTGCCCACAGAAGG | 67.9 |
| 4 | CDH1_Ex4-R | AAAGAAGGATCCCAACACTGG | 54.9 |
| 5 | CDH1_Ex5-F | TTTCTCTGGGAGGGATTTGG | 54.3 |
| 6 | CDH1_Ex5-R | GTAAAACGACGGCCAGTAAGCTCCTCATGTGTTCAGAGC | 66.6 |
| 7 | CDH1_Ex6-F | GGGTCTCAGAGCCTAGGAAGG | 58.6 |
| 8 | CDH1_Ex6-R | GTAAAACGACGGCCAGTTACACAACCTTTGGGCTTGG | 66.7 |
| 9 | CDH1_Ex7-F | GTAAAACGACGGCCAGTGGGCAGAATTGGATTAAGCAG | 66.3 |
| 10 | CDH1_Ex7-R | AACTGACAACTGGCCTAGCAG | 57.2 |
| 11 | CDH1_Ex8-F | GTAAAACGACGGCCAGTATTCTGGTTCCATGTGTTGGG | 66.1 |
| 12 | CDH1_Ex8-R | GCAACTTCACAATCTTGCACC | 55.1 |
| 13 | CDH1_Ex9-F | GCCATGATCGCTCAAATACAC | 54.2 |
| 14 | CDH1_Ex9-R | GTAAAACGACGGCCAGTCAATCTGGGAAAGTCACCCTG | 67 |
| 15 | CDH1_Ex10-F | GTCATGGCAGAAACCACAGTT | 55.8 |
| 16 | CDH1_Ex10-R | GTAAAACGACGGCCAGTGAAGGGAACAGGTGAAAGGAG | 66.9 |
| 17 | CDH1_Ex11-F | CGACCGGCCTATTGTTGG | 56.2 |
| 18 | CDH1_Ex11-R | GTAAAACGACGGCCAGTGCATGTTATTTGGGTGACGG | 66.7 |
| 19 | CDH1_Ex12-F | GTAAAACGACGGCCAGTGTCTGGTGGAAGGCAATGG | 67.9 |
| 20 | CDH1_Ex12-R | CAGAAGGGACAAGGAAGCAAG | 55.8 |
| 21 | CDH1_Ex13-F | GTAAAACGACGGCCAGTGGCTTGCGGGTGTCTTTAG | 67.9 |
| 22 | CDH1_Ex13-R | TGGGAGTCTCTTTCCCACATC | 56.3 |
| 23 | CDH1_Ex14-F | GTAAAACGACGGCCAGTGGCAGCTAGTGGCTGTCTAAC | 68 |
| 24 | CDH1_Ex14-R | TTCAGAGCTGTTTCAAATGCC | 53.9 |
| 25 | CDH1_Ex15-F | GTAAAACGACGGCCAGTAAGGCATCATCCAACCATAATC | 64.6 |
| 26 | CDH1_Ex15-R | GGCAACAAGAGTGAAACTTCG | 54.4 |
| 27 | CDH1_Ex16-F | GTAAAACGACGGCCAGTCACAAGTCTGGGTGCATTGTC | 67.3 |
| 28 | CDH1_Ex16-R | ATCTCAAGGGAAGGGAGCTG | 56.5 |
